# The Predictors of Perceived Barriers and Facilitators of Applying Sepsis Six Guidelines Among Critical Care Nurses

**DOI:** 10.7759/cureus.57355

**Published:** 2024-03-31

**Authors:** Dania Bani Hamad, Mohammad Rababa, Mu’ath I Tanash, Raeda Abuali

**Affiliations:** 1 Department of Applied Sciences/Nursing, Al-Balqa Applied University, Al-Salt, JOR; 2 Department of Adult Health Nursing, Jordan University of Science and Technology, Irbid, JOR; 3 Department of Adult Health Nursing, The Hashemite University, Zarqa, JOR

**Keywords:** nurse, sepsis six guidelines, sepsis, facilitators and barriers, perceived barriers

## Abstract

Background: Sepsis is a life-threatening condition that demands quick and cautious interventions from nurses, as they are the frontline caregivers, so they are essential in recognizing early signs of sepsis, initiating prompt healthcare interventions, and providing comprehensive care to improve patient outcomes. This study aimed to examine the predictors of perceived barriers and facilitators of applying evidence-based sepsis guidelines among critical care nurses.

Methods: This cross-sectional descriptive study was conducted on a convenience sample of 180 nurses working in critical care settings (ICU, critical care unit, ED, burning unit, dialysis unit) at a university hospital. A valid and reliable questionnaire was used to examine the predictors of perceived barriers and facilitators of applying evidence-based sepsis guidelines among critical care nurses.

Results: This study revealed that the main barriers faced by critical care nurses are lack of sepsis recognition during observational rounds and delay in sepsis diagnosis by medical staff. For the most common facilitators of applying Sepsis Six guidelines, the participating nurses reported the presence of a written tool/protocol for sepsis identification and management.

Conclusions: The study emphasized the importance of the presence of evidence-based protocols for sepsis assessment and management and nurses' compliance with guidelines. Ongoing education training for nurses and providing step-by-step written checklists are a cornerstone to improving nurses' knowledge and the practical skills of early identification and management of sepsis.

## Introduction

Sepsis is a global health problem associated with a high mortality rate, especially in critical care units (CCUs) [1]. Recently, sepsis has been defined as a "life-threatening organ dysfunction caused by a dysregulated host response to infection" [2]. Annually, the United States reported at least 900,000 people with sepsis and a 25-30% hospital mortality rate related to sepsis [3]. According to a recent Chinese study in 2022, sepsis was prevalent in 25.5% of ICU patients [4]. Sepsis is a common cause of admission and lengthy hospitalization in Asian healthcare settings. For example, the prevalence of sepsis in the ICU among Asian healthcare settings was 22.4% [5], and in Jordan it was 21% [6]. According to a recent study in 2017, about 49 million cases of sepsis and 11 million deaths were related to sepsis worldwide [7].

Sepsis has serious consequences such as disseminated intravascular coagulopathy [8], acute renal failure [9], encephalopathy [10], and post-sepsis syndrome [11]. According to a French study in 2020, the cost of treating sepsis in the hospital is about 11.400 euros [12]. Therefore, sepsis is a time-sensitive emergency problem that needs rapid identification and management to reduce its morbidity and mortality rates [13].

Critical care nurses are vital in identifying and managing sepsis [6]. Nurses are essential in identifying septic patients by assessing and monitoring early signs and symptoms of sepsis and prompt reporting [6]. Critical care nurses implement many essential interventions for patients with sepsis, including evaluating vital signs, detecting any abnormality, administering antibiotics, providing fluid resuscitation, monitoring lactate levels, and evaluating patient response to treatment [14]. The presence of evidence-based guidelines facilitates the early detection of sepsis by nurses, especially in an emergency department, and assists in appropriately managing patients with sepsis. The Surviving Sepsis Campaign (SSC) developed sepsis one-hour, three-hour, and six-hour bundles focusing on timely identification and rapid management [15]. These bundles are considered the cornerstone of the management of sepsis [16]. The Sepsis Six bundle includes obtaining blood cultures, assessing lactate levels, and administering broad-spectrum antibiotics, oxygen, intravenous fluid, and vasopressor [17]. Many recent studies have shown that compliance with sepsis evidence-based guidelines decreased mortality rate, improved patient outcomes, reduced readmission [15,18,19], and decreased time to initial antibiotics [20]. According to quasi-experimental studies conducted in an emergency department to test the effectiveness of the sepsis bundle guidelines, there was an improvement in the required time to perform sepsis guidelines, and the mortality rate decreased [21]. Many recent studies revealed that the use of evidence-based guidelines led nurses to be autonomous in making decisions related to sepsis management, and the nurses promptly initiated obtaining blood culture and starting resuscitation measures, which led to improved patient outcomes, reduced time of antibiotic demonstration, and reduced mortality rate [22,23].

Recently, a randomized clinical trial study conducted in two medical-surgical intensive care units in California revealed that using a machine sepsis prediction system decreased the length of stay from 13 days to 10 days and the mortality rate by 12.4% [24]. Another recent study has shown that implementing sepsis guidelines reduced the need for ICU admission [25]. Similarly, the nurse’s compliance with the Sepsis Six guidelines leads to decreased sepsis-related complication and mortality rates and improved clinical outcomes [26]. However, low adherence and compliance with applying the Sepsis Six guidelines are still unresolved issues impeding sepsis management due to several barriers [27]. Previous researchers have identified several barriers and facilitators to implementing the Sepsis Six guidelines [20,28]. Identifying the barriers and facilitators is crucial to facilitate and improve the nurse's compliance and adherence to the Sepsis Six guidelines [20]. However, up to date, no study has examined the predictors of these perceived barriers and facilitators among critical care nurses. Accordingly, the study aims to examine the predictors of perceived barriers and facilitators of applying evidence-based Sepsis Six guidelines among critical care nurses.

## Materials and methods

Study design, sample, and settings

A cross-sectional descriptive study was conducted on a convenience sample of 180 nurses who worked in adult critical care settings (ICU, CCU, ED, burning unit, dialysis unit) at a university hospital in Jordan for at least one year. The total number of critical care nurses working in the hospital was 220 nurses, and the sample size targeted by this study was above 80% of the total represented sample. Convenience sampling was used to generate a large sample during a short period. The limit of variation, geographical closeness, and accessibility for the researchers to access the participant's data were other rationales for using convenience sampling. Also, it was impractical within this study to use probability sampling due to high cost, time-consuming, and less convenience for the targeted nurses and researchers within the limited resources. The sample size was determined by G*Power analysis, considering the following parameters: a significant level of 0.05, a statistical power level of 0.8, and a medium effect size, which was enough for this study.

Study instrument

The cross-sectional descriptive study used an online survey from previous research [28,29] and consisted of three parts. The first part includes five questions about demographic data such as age, gender, and marital status. The second part consists of 54 questions about perceived barriers and facilitators of applying sepsis guidelines. Each of the 54 questions consists of two opposing statements rated on a five-point Likert scale ranging from (1) very unimportant to (5) very important [28]. The third part includes seven close-ended questions; two are related to identifying and managing sepsis guidelines, and five are about resources, education, and skills. The face and content validity was done by four nursing scholars holding Ph.D. degrees in critical care nursing who had clinical experience in sepsis assessment and management. They reviewed the questionnaire for clarity, readability, and relevance. According to their feedback, some modifications were recommended and applied in the first version of the questionnaire. Then, they reviewed the modified version for a second time to ensure that the changes suggested were applied by the study researcher and approved the final version. The questionnaire was piloted on 10 nurses, who were excluded from the primary study, to give the necessary feedback and to ensure the clarity of the questionnaire items, which improved the validity of the study instrument before data collection.

Data collection

The researcher met with the nurse manager in the hospital to discuss the eligibility criteria for participation and prepare a list of eligible participant nurses and their emails. All eligible nurses have been invited to take part in the study by sending an invitation e-mail containing the aims of the study, brief explanations of the study, and the rights and responsibilities of the participants. A reminder e-mail was used two weeks after the initial communication with the participants to improve the response rate. All nurses who replied to our invitation received another e-mail containing the consent form and the study survey. Data collection was carried out in January 2024.

Ethical consideration

Ethical approval (2024/2023/3/17) for the study was received from the Institutional Review Board (IRB) at Balqa Applied University and the study setting. Written informed consent was obtained from the participating nurses. The participants were told they could withdraw from the study at any time. The privacy and confidentiality of collected data were assured throughout the study. We encouraged the potential participants to declare any conflict of interest they may have before including them in the study.

Statistical data analysis

The mean and standard deviation were used to describe continuous measured variables. The frequency and percentages were used to describe categorically measured variables. The Kolmogorov-Smirnov (KS) statistical test of normality and the histogram were used to assess the statistical normality of metric variables assumption. The bivariate Pearson's correlation test assessed the correlations between metric-measured variables. The multivariable linear regression analysis was applied to determine the statistical significance of possible predictors for nurses' perceived barriers and facilitators of applying Sepsis Six bundles. The association between the predictor-independent variables with the analyzed outcomes in the multivariable linear regression analysis was expressed as an unstandardized beta coefficient with its associated 95% confidence intervals. The latest version of the IBM SPSS statistical computing test (IBM Corp., Armonk, NY) was used for the statistical analysis, and the alpha significance level was considered at 0.050.

## Results

A total of 180 critical care nurses enrolled in the study and completed and returned the study survey. Table 1 displays the nurses' sociodemographic characteristics and working and professional factors. The findings showed that 52.8% of the sample were male, and 23.9% were unmarried. Around 34% of nurses had experience between one and four years, and 24.4% held a master's degree in nursing. Of the nurses, 21.1% worked in CCU, 22.2% in ER, 35.6% in surgical-medical ICU, and 21.1% in other units (dialysis and burn units).

**Table 1:** Descriptive analysis of the nurses' sociodemographic characteristics and working and professional factors.

	Frequency	Percentage
Sex		
Male	95	52.8
Female	85	47.2
Marital status		
Never married	43	23.9
Ever married	137	76.1
Experience years		
1-4 years	62	34.4
5-10 years	84	46.7
>=11 years	34	18.9
Working department		
Critical care unit (CCU)	38	21.1
Emergency room (ER)	40	22.2
Intensive care unit (ICU)	64	35.6
Other (dialysis unit, burn)	38	21.1
Educational level		
University degree/diploma	136	75.6
Higher studies	44	24.4

Perspectives on reasons for delayed sepsis identifications

**Figure 1:**
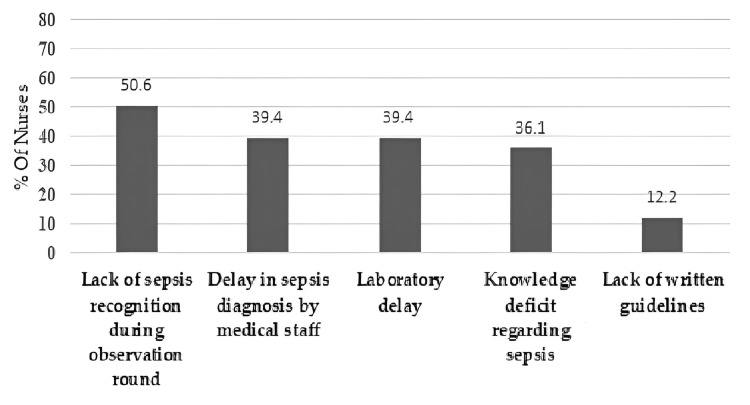
Nurses' perceived sources of delay sources for sepsis identification.

The nurses' experiences with sepsis management were also measured via several questions that measured their opinions about delays in sepsis identification and management. The nurses were asked to indicate the most common causes of delay in identifying patients with sepsis. These causes were lack of sepsis recognition and identification during observation rounds by doctors, delay in sepsis diagnosis by physicians, delay in sending the results from the lab, knowledge deficit regarding the signs, symptoms, and treatment of sepsis, and lack of written guidelines for nurses to implement intervention for patients with sepsis. The analysis findings showed that most of the nurses (50.6%) believed lack of sepsis recognition during observation rounds may delay sepsis identification. Figure 1 shows the remaining prevalence of reasons for delayed sepsis identification according to nurses’ perspectives.

Perspectives on reasons for delayed sepsis treatment

**Figure 2:**
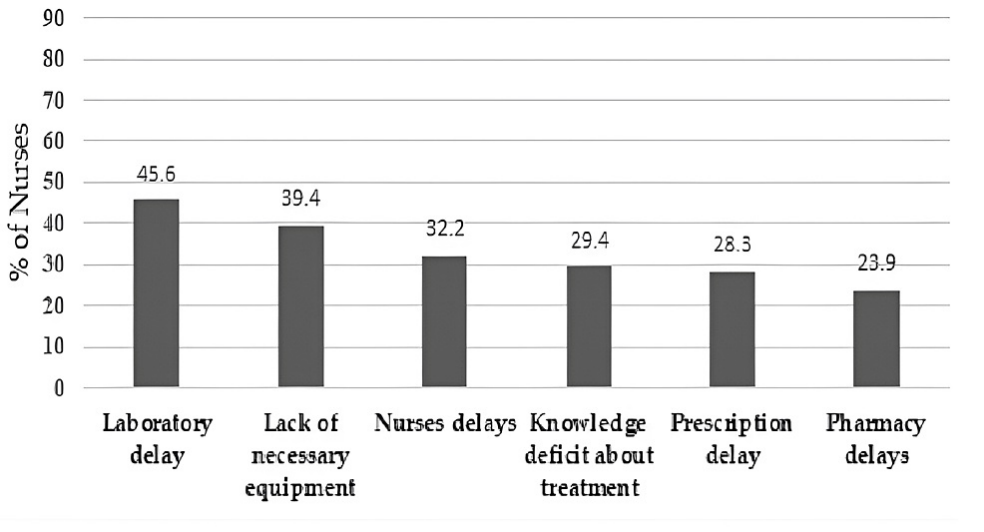
Nurses' perceived causes of sepsis treatment delay sources.

Furthermore, the findings showed that the majority of nurses (45.6%) believed that laboratory delays in sending the results may contribute to delayed sepsis treatment. The second reason is the lack of necessary equipment to diagnose and treat sepsis, such as blood cultures, antibiotics, and beds. The third reason was nurses' delay in initiating treatment and managing patients with sepsis. According to nurses' perspectives, Figure 2 shows the prevalence of remaining reasons for delayed sepsis treatment.

Description of nurses’ perceived barriers and facilitators

The findings also showed that 61.1% of the nurses had previous sepsis identification and management training. Another 50.6% of them advised that the courses on sepsis management they have undertaken were satisfactory. However, the analysis findings showed that 67.2% of the nurses were aware that serum lactate test results can influence/guide septic patients' management. Also, most of the nurses (77.8%) agreed that sepsis treatments were available at their point-of-care areas (like IV fluids, oxygen, and first-line antibiotics). Also, most of the nurses (81.7%) had confirmed the availability of sepsis investigations at their point-of-care areas (like blood lactate tests, blood culture, and urinary catheters and containers). The detailed descriptive analysis is outlined in Table 2.

**Table 2:** Descriptive analysis of the nurses' perceptions about the delays in sepsis management, sepsis management experiences, and areas of improvement in sepsis.

	Frequency		Percentage
What do you consider to be the greatest cause of delay in the identification of sepsis?			
1. Delay in sepsis diagnosis by medical staff		71	39.4
2. Lack of sepsis recognition during the observation round		91	50.6
3. Laboratory delay		71	39.4
4. Knowledge deficit regarding sepsis		65	36.1
5. Lack of written guidelines		22	12.2
What do you consider to be the greatest cause of delay in the treatment of sepsis?			
1. Pharmacy delays		43	23.9
2. Nurses' delays		58	32.2
3. Prescription delay		51	28.3
4. Lack of necessary equipment		71	39.4
5. Laboratory delay		82	45.6
6. Knowledge deficit regarding appropriate treatment		53	29.4
What areas of the sepsis education program do you believe can be improved?			
1. Identifying septic patients		96	53.3
2. Applying the sepsis pathway		95	52.8
3. Practical skills (e.g., cannulation, venipuncture, and applying the sepsis pathway)		88	48.9
4. Other areas		23	12.8
Have you had training regarding the identification and management of sepsis?			
No		70	38.9
Yes		110	61.1
If you had a course previously, was it sufficient/satisfactory?			
No		89	49.4
Yes		91	50.6
Do you know how a serum lactate test/result can be used to influence the management of a septic patient?			
No		59	32.8
Yes		121	67.2
Are the sepsis treatments available at the point of care (fluid, oxygen, and first-line antibiotics)?			
No		40	22.2
Yes		140	77.8
Are the sepsis investigations available at the point of care (blood lactate, blood culture, and urinary catheterization)?			
No		33	18.3
Yes		147	81.7

Predictors of nurses’ perceived facilitators of sepsis management

The multivariable linear regression analysis (MLRA) was used to examine the predictors of nurses' perceived facilitators of Sepsis Six performance bundle (SSPB) implementation at the workplace to understand what explains why the nurses perceived less or more importance to the Sepsis Six performance at the workplace. The yielded analysis findings (Table 3) showed that the nurses' sex, marital status, educational level, and experience years were not associated significantly with their overall mean perceived SSPB importance score (p-value > 0.050). However, nurses who believed that physicians delayed diagnosis of sepsis patients had significantly higher overall mean perceived SSPB importance scores compared to nurses who disagreed, with a beta coefficient of 0.312 and p-value of 0.006. Also, nurses who perceived a lack of written guidelines at the workplace may delay sepsis diagnoses had a significantly higher overall mean perceived SSPB importance score compared to those who disagreed, with a beta coefficient of 0.498 and p-value of 0.002. Not only that but also the nurses who believed that sepsis investigations and lab tests were available at the point of care had perceived significantly higher overall mean perceived SSPB importance scores compared to nurses who reported the absence of the investigations, with a beta coefficient of 0.321 and p-value of 0.016. Moreover, the analysis model showed that the nurses who perceived knowledge deficit as a cause for sepsis diagnosis delay had perceived the SSPB as significantly more important than those who did not perceive it (beta coefficient = 0.447; p-value < 0.001).

**Table 3:** Multivariable linear regression analysis of nurses’ perceived facilitators of Sepsis Six performance bundle (SSPB) implementation (N = 180). Dependent outcome variable: Nurses mean perceived overall importance of Sepsis Six. Model R-squared = 0.294, adjusted R-squared = 0.243. Model overall statistical significance: f (12, 167) = 5.80, p-value < 0.001.

		95.0% CI for beta coefficient		
	Unstandardized beta coefficients	Lower bound	Upper bound	p-value
Constant	3.095	2.462	3.727	<0.001
Gender: female vs. male	0.062	-0.154	0.277	0.573
Marital status: never married	-0.224	-0.479	0.031	0.084
Working unit	-0.046	-0.143	0.050	0.344
Experience years	-0.025	-0.188	0.138	0.761
Education level	0.137	-0.098	0.371	0.252
Perceived knowledge	0.122	0.020	0.224	0.019
Perceived belief in capability	0.154	-0.020	0.329	0.083
Perceived belief in consequences	-0.177	-0.336	-0.018	0.030
Delay in sepsis diagnosis by medical staff	0.312	0.092	0.531	0.006
Lack of written guidelines	0.498	0.179	0.818	0.002
Availability of sepsis investigation	0.321	0.061	0.580	0.016
Knowledge deficit	0.447	0.239	0.654	<0.001

Predictors of nurses’ perceived barriers to sepsis management

Also, to arrive at better insight on what may explain why the nurses had perceived less or more barriers to implementing the SSPB at their workplace, the MLRA was applied as well to regress their overall mean perceived SSPB implementing barriers score against their sociodemographic characteristics and work and professional-related factors and perceptions. The yielded multivariable analysis findings (Table 4) suggested that the nurses' sex, marital status, working units, experience years, and educational level did not correlate significantly with their overall mean perceived SSPB implementing barriers score (p-value > 0.050). However, the analysis findings showed that the nurses' overall mean perceived SSPB importance score was positively and significantly associated with their overall mean perceived SSPB implementing barriers score. Interestingly, the analysis findings showed that the nurses who agreed that physicians might delay the sepsis diagnoses had a significantly lower overall mean perceived SSPB implementing barriers score than those who disagreed that physicians might cause sepsis diagnosis delay (beta coefficient = -0.459; p-value < 0.001). Also, the nurses who had perceived a lack of sepsis recognition during observation rounds may delay sepsis had perceived significantly lower overall mean perceived SSPB implementing barriers score compared to nurses who did not experience such lack of sepsis recognition (beta coefficient = -0.482; p-value < 0.001). Not only that but also the nurses who perceived lack of written guidelines may hinder sepsis diagnoses had perceived significantly lower overall mean perceived SSPB implementing barriers score compared to those who disagreed (beta coefficient = -0.508; p-value = 0.004).

**Table 4:** Multivariable linear regression analysis of nurses' means perceived overall barriers of Sepsis Six barriers score (N = 180). Dependent outcome variable: Nurses mean perceived overall barriers of Sepsis Six. Model R-squared = 0.248, adjusted R-squared = 0.207. Model overall statistical significance: f (9, 169) = 5.91, p-value < 0.001.

		95.0% CI for beta coefficient		
	Unstandardized beta coefficients	Lower bound	Upper bound	p-value
Constant	3.002	2.233	3.771	<0.001>
Gender = female vs. male	-0.210	-0.441	0.021	0.074
Marital status: never married	-0.023	-0.290	0.245	0.866
Working unit	-0.096	-0.200	0.007	0.068
Experience years	0.005	-0.170	0.180	0.953
Education level	0.181	-0.074	0.435	0.163
Perceived importance of Sepsis Six performance	0.208	0.051	0.364	0.010
Delay in sepsis diagnosis by medical staff	-0.459	-0.690	-0.228	<0.001>
Lack of sepsis recognition during observation round	-0.482	-0.695	-0.269	<0.001>
Lack of written guidelines	-0.508	-0.849	-0.166	0.004

## Discussion

This is the first study in Jordan to examine the predictors of perceived barriers and facilitators of applying Sepsis Six guidelines among critical care nurses. Our study found that nurses' sociodemographic characteristics did not influence their perceived barriers and facilitators of applying sepsis guidelines. Our study revealed that lack of sepsis recognition during observational rounds is considered the most significant cause of delayed identification of sepsis. Similarly, Breen’s study was conducted on a convenience sample from nurses and junior doctors who work in an emergency department [29]. This study also found that laboratory delay is considered the most significant cause of delayed management and treatment of sepsis. This is consistent with Breen’s study, which found nursing delay, knowledge deficit, and laboratory delay are the major causes of delayed management of sepsis [29].

Our finding revealed the importance of a written tool or protocol to guide sepsis assessment and management. Consistent with previous studies, applying sepsis protocols/guidelines was highly effective in the early identification and management of sepsis and improved nurses' compliance with them [30]. Also, regular use of sepsis guidelines makes it easier to remember the sepsis involved and improves nurses' performance [15].

Several studies revealed that a lack of knowledge and unfamiliarity with the six guidelines for sepsis can lead to delayed identification of patients with sepsis [20,28,29]. Also, the current study revealed a lack of nurses' knowledge regarding sepsis, which is considered one of the main barriers to applying sepsis guidelines. Previous studies showed that Jordanian nurses have poor knowledge regarding sepsis identification and management, which may be attributed to several causes, including the nursing schools in Jordan do not focus on sepsis identification and management [6]. Moreover, inadequate ongoing education programs for nursing staff on sepsis identification and management could be another factor [6]. Also, the finding of the current study that nursing delay is considered one of the leading causes of delaying sepsis management and treatment was consistent with the finding of Burney et al. [31] that the shortage of nursing is a primary barrier to the management of sepsis.

The current study revealed that the nurses are aware of the lactate level influence and guide sepsis identification and management. This is inconsistent with a previous Canadian study that showed that emergency nurses had a low level of knowledge and awareness about the effect of lactate level on early identification and management of sepsis [32]. Maybe because ongoing education programs can improve nurses’ awareness about the impact of the lactate level on early identification and management of sepsis. Our study revealed that sepsis investigation and treatment are available at the point of care. This result suggests that the nursing and medical team can identify septic patients and initiate prompt and urgent management. These findings, consistent with the previous studies, show the lack of necessary equipment can delay sepsis identification and management and then increase the morbidity and mortality rate related to sepsis [28,31]. This study found the significant areas of sepsis education programs that nurses believe can be improved, including identifying sepsis patients and identifying sepsis pathways consistent with previous studies that found the importance of educating nurses about the early signs and symptoms of sepsis to facilitate early identification of septic patients [6,33]. Also, the nurses must improve their practical skills, including cannulation, blood culture, administering antibiotics, and other skills. This study emphasized the importance of ongoing education training for nurses, especially in identifying sepsis and applying the sepsis pathway. Consistent with the previous study conducted on nurses and junior doctors working in an emergency department, the doctors need further education in an applied sepsis pathway. Still, nurses need further assessment of practical issues [29]. Another quasi-experimental study conducted on 40 nurses in neurosurgical wards and ICU found that education sessions can improve nurses’ knowledge about sepsis guidelines and the quality of care for septic patients [34]. Another prospective study aimed to assess adherence and compliance with sepsis bundles after the education program revealed improved adherence and compliance and reduced children's hospital stays related to sepsis [35]. Also, the education program can improve nurses’ competence regarding sepsis identification and management [36].

Limitations of the study

This study was conducted only in one geographic area due to limited resources and a restricted timeframe, which may limit the generalizability of the data. Using a non-probability convenience sample may cause selection bias, affect the representativeness of the participants, and threaten internal validity. Employing a more rigorous sampling method, such as random sampling, could enhance the validity of the study. The study was conducted in only one geographic area, which may limit the generalizability of the findings. Future studies including participants from multiple regions could provide a more comprehensive understanding of the predictors of perceived barriers and facilitators of applying sepsis guidelines among critical care nurses. Also, there are other limitations regarding using online questionnaires; some nurses are unfamiliar with them and face technical problems. Although the researcher sent multiple reminders in the email on how the students should fill out the survey, using an online questionnaire may have led the participants to offer less than accurate responses, which could have an impact on the reliability of the findings.

Clinical implication and recommendation

The findings of this study could help hospital managers develop ongoing education sessions about sepsis management for both physicians and nurses. Also, our findings could be used in developing specific and quick written tools/checklists to facilitate early assessment and management of sepsis, which may improve nurses' compliance and adherence to sepsis guidelines. Further mixed method and interventional studies are needed to assess the barriers and facilitators of applying sepsis guidelines in critical care settings. In future studies, a large sample size, including multidisciplinary participants and more than one region, is recommended to improve the findings' generalizability and the data's reliability and validity. Future studies are advised to conduct experimental studies using random samples to enhance the generalizability of the findings and eliminate the selection bias. Also, future qualitative studies are recommended to extensively identify the barriers and facilitators of sepsis management. The replication of the study in multi-geographical areas or multi-setting is also recommended in the future.

Innovative and practical tools should be developed to improve the understanding and implementation of Sepsis Six guidelines by physicians and nurses. For example, developing oriented booklets containing helpful information for professionals on effectively implementing Sepsis Six guidelines is recommended. Future interventional studies to examine the effectiveness of these practical tools in improving the treatment fidelity of sepsis among critical care nurses are recommended. In Jordan, as in many developing countries, there is an urgent need to involve expert professional representatives in future reviews of national health guidelines to ensure that their perspectives are incorporated to achieve an evidence-based approach to sepsis management. Furthermore, organizational policies on health education, the detection of sepsis, and the implementation of Sepsis Six guidelines should be simple, straightforward, and accessible to all professionals without technical or material obstacles.

## Conclusions

Early identification and management of sepsis is a critical issue to decrease the morbidity and mortality rate related to sepsis. The presence of evidence-based sepsis guidelines can facilitate and improve the early identification and management of sepsis. This study identified various barriers faced by critical care nurses in applying Sepsis Six guidelines, including lack of sepsis recognition during observational rounds, delay in sepsis diagnosis by physicians, laboratory delay in sending the results, and lack of necessary equipment and lack of tool/checklists that guide critical care nurses to early detection and management of sepsis. Also, the study identified multiple nurses’ perceived facilitators of applying Sepsis Six guidelines. These facilitators include enhanced nurses’ knowledge of sepsis, awareness of sepsis consequences, the importance of written evidence-based sepsis guidelines, prompt sepsis diagnosis, and available sepsis investigations. The study suggested that providing continuous education programs related to sepsis identification for healthcare providers, developing further sepsis investigation tools, and using them as a part of the protocol and policies of healthcare settings would facilitate and improve the early identification and management of sepsis. Thus, improving the quality of hospital care and reducing morbidity and mortality rates due to sepsis.

## References

[1] Gauer R, Forbes D, Boyer N (2020). Sepsis: diagnosis and management. Am Fam Physician.

[2] Singer M, Deutschman CS, Seymour CW (2016). The third international consensus definitions for sepsis and septic shock (Sepsis-3). JAMA.

[3] Fleischmann C, Scherag A, Adhikari NK (2016). Assessment of global incidence and mortality of hospital-treated sepsis. Current estimates and limitations. Am J Respir Crit Care Med.

[4] Lei S, Li X, Zhao H, Xie Y, Li J (2022). Prevalence of sepsis among adults in China: a systematic review and meta-analysis. Front Public Health.

[5] Li A, Ling L, Qin H (2022). Epidemiology, management, and outcomes of sepsis in ICUs among countries of differing national wealth across Asia. Am J Respir Crit Care Med.

[6] Rababa M, Bani-Hamad D, Hayajneh AA, Al Mugheed K (2022). Nurses' knowledge, attitudes, practice, and decision-making skills related to sepsis assessment and management. Electron J Gen Med.

[7] Fleischmann-Struzek C, Mellhammar L, Rose N (2020). Incidence and mortality of hospital- and ICU-treated sepsis: results from an updated and expanded systematic review and meta-analysis. Intensive Care Med.

[8] Iba T, Connors JM, Nagaoka I, Levy JH (2021). Recent advances in the research and management of sepsis-associated DIC. Int J Hematol.

[9] Skube SJ, Katz SA, Chipman JG, Tignanelli CJ (2018). Acute kidney injury and sepsis. Surg Infect (Larchmt).

[10] Chen J, Shi X, Diao M, Jin G, Zhu Y, Hu W, Xi S (2020). A retrospective study of sepsis-associated encephalopathy: epidemiology, clinical features and adverse outcomes. BMC Emerg Med.

[11] Mostel Z, Perl A, Marck M (2019). Post-sepsis syndrome - an evolving entity that afflicts survivors of sepsis. Mol Med.

[12] Dupuis C, Bouadma L, Ruckly S (2020). Sepsis and septic shock in France: incidences, outcomes and costs of care. Ann Intensive Care.

[13] Harley A, Johnston AN, Denny KJ, Keijzers G, Crilly J, Massey D (2019). Emergency nurses' knowledge and understanding of their role in recognising and responding to patients with sepsis: a qualitative study. Int Emerg Nurs.

[14] Kleinpell R, Blot S, Boulanger C, Fulbrook P, Blackwood B (2019). International critical care nursing considerations and quality indicators for the 2017 Surviving Sepsis Campaign guidelines. Intensive Care Med.

[15] Frank HE, Evans L, Phillips G (2023). Assessment of implementation methods in sepsis: study protocol for a cluster-randomized hybrid type 2 trial. Trials.

[16] Nunnally ME, Ferrer R, Martin GS (2021). The Surviving Sepsis Campaign: research priorities for the administration, epidemiology, scoring and identification of sepsis. Intensive Care Med Exp.

[17] McVeigh SE (2020). Sepsis management in the emergency department. Nurs Clin North Am.

[18] Arabi YM, Alsaawi A, Al Zahrani M (2021). Electronic early notification of sepsis in hospitalized ward patients: a study protocol for a stepped-wedge cluster randomized controlled trial. Trials.

[19] Herrán-Monge R, Muriel-Bombín A, García-García MM (2019). Epidemiology and changes in mortality of sepsis after the implementation of Surviving Sepsis Campaign guidelines. J Intensive Care Med.

[20] Reich EN, Then KL, Rankin JA (2018). Barriers to clinical practice guideline implementation for septic patients in the emergency department. J Emerg Nurs.

[21] Delawder JM, Hulton L (2020). An interdisciplinary code sepsis team to improve sepsis-bundle compliance: a quality improvement project. J Emerg Nurs.

[22] Proffitt RD, Hooper G (2020). Evaluation of the (qSOFA) tool in the emergency department setting: nurse perception and the impact on patient care. Adv Emerg Nurs J.

[23] Roney JK, Whitley BE, Long JD (2020). Implementation of a MEWS-Sepsis screening tool: transformational outcomes of a nurse-led evidence-based practice project. Nurs Forum.

[24] Shimabukuro DW, Barton CW, Feldman MD, Mataraso SJ, Das R (2017). Effect of a machine learning-based severe sepsis prediction algorithm on patient survival and hospital length of stay: a randomised clinical trial. BMJ Open Respir Res.

[25] Venkatesh B, Schlapbach L, Mason D (2022). Impact of 1-hour and 3-hour sepsis time bundles on patient outcomes and antimicrobial use: a before and after cohort study. Lancet Reg Health West Pac.

[26] Keeley A, Hine P, Nsutebu E (2017). The recognition and management of sepsis and septic shock: a guide for non-intensivists. Postgrad Med J.

[27] Mosavianpour M, Collett J, Sarmast H, Kissoon N (2016). Barriers to the implementation of sepsis guideline in a Canadian pediatric tertiary care centre. J Nurs Educ Pract.

[28] Roberts N, Hooper G, Lorencatto F, Storr W, Spivey M (2017). Barriers and facilitators towards implementing the Sepsis Six care bundle (BLISS-1): a mixed methods investigation using the theoretical domains framework. Scand J Trauma Resusc Emerg Med.

[29] Breen SJ, Rees S (2018). Barriers to implementing the Sepsis Six guidelines in an acute hospital setting. Br J Nurs.

[30] McCaffery M, Onikoyi O, Rodrigopulle D, Syed A, Jones S, Mansfield L, Krishna MG (2016). Sepsis-review of screening for sepsis by nursing, nurse driven sepsis protocols and development of sepsis hospital policy/protocols. Nurs Palliat Care.

[31] Burney M, Underwood J, McEvoy S, Nelson G, Dzierba A, Kauari V, Chong D (2012). Early detection and treatment of severe sepsis in the emergency department: identifying barriers to implementation of a protocol-based approach. J Emerg Nurs.

[32] Storozuk SA, MacLeod ML, Freeman S, Banner D (2019). A survey of sepsis knowledge among Canadian emergency department registered nurses. Australas Emerg Care.

[33] Bentley J, Henderson S, Thakore S, Donald M, Wang W (2016). Seeking sepsis in the emergency department- identifying barriers to delivery of the Sepsis 6. BMJ Qual Improv Rep.

[34] Nakiganda C, Atukwatse J, Turyasingura J, Niyonzima V (2022). Improving nurses’ knowledge on sepsis identification and management at Mulago National Referral Hospital: a quasi experimental study. Nurs Res Rev.

[35] Fernández-Sarmiento J, Carcillo JA, Salinas CM, Galvis EF, López PA, Jagua-Gualdrón A (2018). Effect of a sepsis educational intervention on hospital stay. Pediatr Crit Care Med.

[36] Delaney MM, Friedman MI, Dolansky MA, Fitzpatrick JJ (2015). Impact of a sepsis educational program on nurse competence. J Contin Educ Nurs.

